# The Common Task

**Published:** 1907-10-19

**Authors:** 


### THE COMMON TASK.

Correspondence and Queries for this section should be sent to the Editor of The Hospital, 28 Southampton Street, Strand, London, and marked "Nursing Administration."

### Hygiene in Elementary Schools.

We note with much satisfaction the increased importance attached by the London County Council to the study of hygiene by teachers in elementary schools. Among the classes and lectures provided this winter for the teachers are several courses which are likely to have an effect for good in smoothing the work of the nurses appointed to visit the schools. There is an excellent course on Hygiene by Professor Simpson and Dr. Somerville, an elementary course on Bacteriology by Miss Tchaykovsky, dealing with infective processes, and an admirably thought-out course dealing on hygiene in relation to physical education. Teachers who have had the opportunity of attending such courses are likely to be of the greatest service to the inspecting nurses, who are now doing such valuable work in elementary schools. The teachers have it in their power to minimise the nurse's influence by contemptuous dismissal of precautions and good counsel as mere fads, and anything which opens their eyes to the real meaning of the work accomplished by medical officer and nurse reacts with unsuspected force on the personal habits of the children.

### Pig-Wash.

Much may be learned by the inquiring guardian or hospital superintendent who will have the energy to arise in the morning and examine into the remarkable things which find an entrance into the pig-tub, and are fetched away before the world is awake. The contractor for waste products is, perhaps, a necessary evil in institution economy. He certainly makes a profit out of all reason when the authorities are not alive to the value of what they sell, and how many are there who study to attain this knowledge? Meat, bread, vegetables, milk, beef-tea, fat, bones, dripping, odds and ends of pudding, porridge, and countless ingredients which have good food value, are included among the assorted articles which are collected with anxious care by the contractor, and thankfully parted with by the institution without a moment's misgiving. Our contention is that much of this ought never to be sold at all, but should be used up in the kitchen. For the remainder a price far exceeding what is usually paid ought to be obtained. It is the matron's business to know exactly what weight of waste material is unavoidably produced in the course of a day, or week, and to have it removed under supervision. Were the heads of large institutions to take the trouble to go into this matter, not deeming it beneath their dignity to lend it their personal attention, we believe that the money gained and saved together over this matter of pig-wash would open many beds now "unavoidably closed for want of funds."

### New Rules of the Central Midwives Board.

The recent edition of the rules framed by the Central Midwives Board contains a careful revision of the regulations, "supervising and restricting within due limits the practice of midwives." In particular we note that the rules prescribing "conditions in which medical help must be sent for" have been rendered much more precise, the form of procedure being now plain and easy to the dullest intelligence. The duty of reporting to the local supervising authority was under the former rules involved in an obscurity which puzzled even experienced women. It is matter for regret that the rule which prohibited midwives from laying out the dead has been relaxed, not only in the case of infants, but also in the case of patients dying of non-infectious illness, provided the nurse is not attending a midwifery case at the time. The old rule was a great protection to district midwives, and we believe it is for their interests in village and district work that it should not be abrogated by their superintendents. The vexed question of the doctor's fee does not, of course, fall within the scope of these rules. It is, however, a matter of some urgency, especially in view of recent advances in scientific midwifery. Supposing that the doctor is compelled to use the serum treatment for a case thrown on his hands by a midwife, is he to bear the whole expense of this himself in the event of the patient's husband being unable to pay? And how can the labourer earning some 14s. or 16s. a week produce the necessary funds? This is a problem which cries for solution.

### The Choice of Assistant Nurses.

There is no point on which a committee is apt to be more tenacious than that of selecting the subordinate staff in institutions. The kind of patronage which is involved in making appointments is peculiarly dear to the mind of man, also to the mind of woman. Sometimes the matron is consulted, sometimes her opinion is disregarded, but it is seldom that she is allowed, as in our opinion she ought to be, the casting-vote. Boards of guardians are peculiarly liable to show distrust towards their matrons in the matter of selecting probationers and assistants. There is no doubt that the advice of a few business men or women is of the greatest assistance in sifting applications, but in the last resort it is the matron whose work will be made or marred by the persons selected, and it is far more to her interest to keep clear of all considerations which tend to bias a choice than it is to that of any of the committee. Few matrons rise to that position without acquiring a shrewd insight into character, and it is not wise to disregard the feeling which prompts an experienced woman to say, "This will do," or "This will not do," even though she may be quite unable to translate her verdict into solid reasons. Lastly, all things being equal, there is a trait in many estimable people which makes them inclined to make the best of those whom they have selected, and the worst of those who have been foisted upon them. It is worth while to run counter to the natural liking of a keen administrator to choose her own instruments by insisting on a point of little moment to anyone but her whose prerogative is at stake?

